# Model‐based dynamic off‐resonance correction for improved accelerated fMRI in awake behaving nonhuman primates

**DOI:** 10.1002/mrm.29167

**Published:** 2022-01-26

**Authors:** Mo Shahdloo, Urs Schüffelgen, Daniel Papp, Karla L. Miller, Mark Chiew

**Affiliations:** ^1^ Wellcome Centre for Integrative Neuroimaging Department of Experimental Psychology University of Oxford Oxford UK; ^2^ Wellcome Centre for Integrative Neuroimaging FMRIB Nuffield Department of Clinical Neurosciences University of Oxford Oxford UK; ^3^ NeuroPoly Lab Electrical Engineering Department Polytechnique Montréal Montreal Canada

**Keywords:** dynamic off‐resonance, EPI, fMRI, nonhuman primates, simultaneous multislice

## Abstract

**Purpose:**

To estimate dynamic off‐resonance due to vigorous body motion in accelerated fMRI of awake behaving nonhuman primates (NHPs) using the echo‐planar imaging reference navigator, in order to attenuate the effects of time‐varying off‐resonance on the reconstruction.

**Methods:**

In NHP fMRI, the animal’s head is usually head‐posted, and the dynamic off‐resonance is mainly caused by motion in body parts that are distant from the brain and have low spatial frequency. Hence, off‐resonance at each frame can be approximated as a spatially linear perturbation of the off‐resonance at a reference frame, and is manifested as a relative linear shift in k‐space. Using GRAPPA operators, we estimated these shifts by comparing the navigator at each time frame with that at the reference frame. Estimated shifts were then used to correct the data at each frame. The proposed method was evaluated in phantom scans, simulations, and in vivo data.

**Results:**

The proposed method is shown to successfully estimate spatially low‐order dynamic off‐resonance perturbations, including induced linear off‐resonance perturbations in phantoms, and is able to correct retrospectively corrupted data in simulations. Finally, it is shown to reduce ghosting artifacts and geometric distortions by up to 20% in simultaneous multislice in vivo acquisitions in awake‐behaving NHPs.

**Conclusion:**

A method is proposed that does not need sequence modification or extra acquisitions and makes accelerated awake behaving NHP imaging more robust and reliable, reducing the gap between what is possible with NHP protocols and state‐of‐the‐art human imaging.

## 1 INTRODUCTION

Nonhuman primates (NHPs) serve as useful models for understanding the human brain due to the many functional and structural parallels between the two species.^1^ However, NHP brain imaging involves unique challenges relative to human neuroimaging. For example, the macaque neocortex is 15 times smaller in volume, and is 25% thinner compared to the human brain.^2^ These considerable scale differences necessitate imaging smaller voxels in order to achieve similar levels of spatial delineation that inherently reduces the signal to noise ratio (SNR), which is further reduced when undersampling is used to accelerate data acquisition. As a remedy, simultaneous multislice (SMS) imaging^3^, ^4^ using bespoke multichannel receive coils ^5^ has been recently employed to enhance statistical power by retaining the temporal degrees of freedom while accelerating the scans in anesthetized NHPs.^6^, ^7^

**FIGURE 1:**
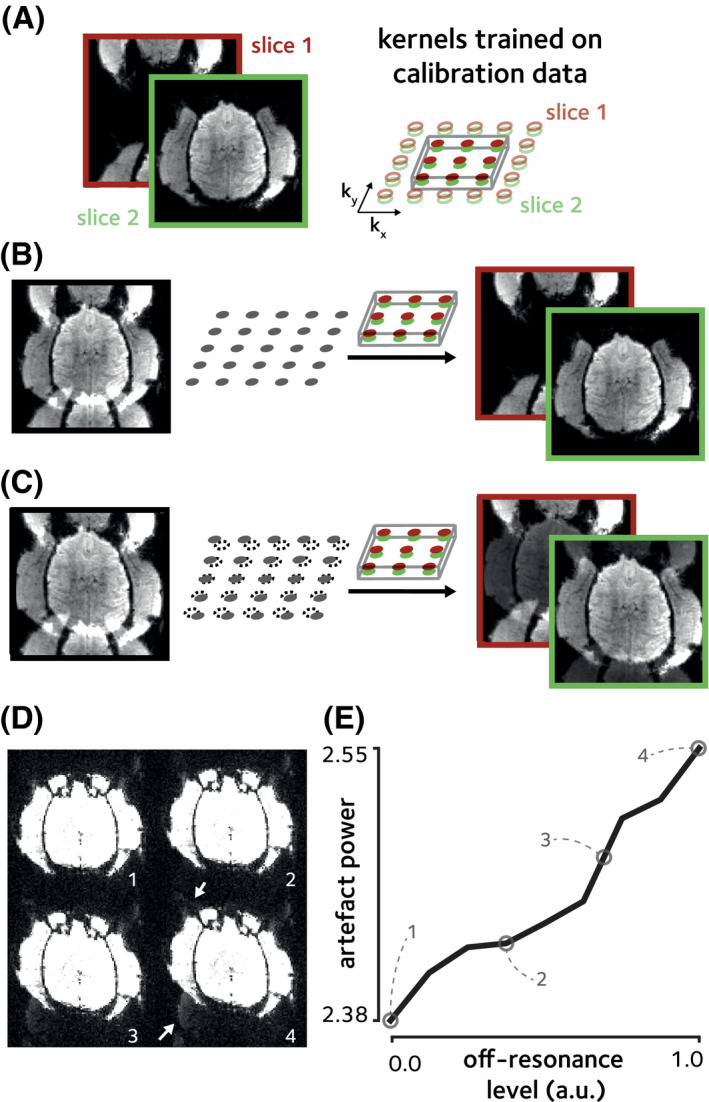
Ghosting artifacts and geometric distortion due to off‐resonance. Simultaneous multislice accelerated data (MB = 2, with CAIPI shifts^33^) were simulated using a single data‐frame from fully sampled in vivo macaque data. Simulated spatially linear off‐resonance was added. (A) The fully sampled data were used to train split‐slice GRAPPA^34^ kernels. (B) In the absence of off‐resonance perturbations, these trained kernels could be used to completely separate the aliased slices. (C) Off‐resonance perturbation, however, results in inconsistency of the data to the trained kernels which leads to aliasing artifacts manifested as Nyquist ghosts as well as geometric distortion. (D) Increasing the off‐resonance level (indicated by numbers) enhances the level of ghosting artifacts and geometric distortion. Here, the display range is saturated to enhance the visibility of artifacts (indicated by white arrows). (E) Artifact power, taken as the $\ell_{2}$ norm of the background values, versus off‐resonance level. Values corresponding to off‐resonance levels in (D) are marked by empty circles. Artifact power monotonically increases by increasing the levels of off‐resonance

Echo‐planar imaging (EPI) in awake behaving NHPs is further complicated due to vigorous motion in the behaving animal’s body, hands, jaw, and facial musculature.^8^ These movements that inevitably occur, even after mechanical head stabilization, induce unpredictable $B_{0}$ off‐resonance changes that are orders of magnitude larger than those induced by respiration or cardiac activity in anesthetized animals.^9^, ^10^ While geometric distortion due to these dynamic effects can be partly addressed in image‐based post‐processing,^11^ off‐resonance can render the acquired data inconsistent with the reference calibration data used for unaliasing reconstruction.^12^ This inconsistency could result in additional substantial Nyquist ghosting and residual aliasing artifacts that degrade image quality and potentially obscure the signals of interest. Unlike correcting for distortions caused by static field inhomogeneity, these dynamic reconstruction artifacts are not addressable using image‐based post‐processing or global ghost correction techniques (Figure 1), for a number of reasons. Since the significant off‐resonant effects result in images with considerable ghosting and aliasing, reconstructed images themselves cannot be used to generate reliable field maps using phase‐ or magnitude‐based methods. Furthermore, methods based on interleaved blip up/down phase‐encoding directions cannot be used to estimate dynamic field maps since it is not guaranteed that the off‐resonance remain the same between readouts in the presence of vigorous motion in NHPs.^13^ Hence, accurate estimation of dynamic off‐resonance prior to image reconstruction is crucial for high‐quality accelerated fMRI imaging of awake behaving NHPs.

Several approaches have been proposed to estimate dynamic off‐resonance in human neuroimaging using field sensors,^14^, ^15^, ^16^ multi‐echo sequences.^17^, ^18^, ^19^, ^20^, or extra navigators.^21^, ^22^, ^23^, ^24^ In the scope of awake behaving NHP fMRI, these previous works are limited by requiring complicated additional hardware, compromising temporal resolution to accommodate multiple echoes, or requiring customized sequences. Alternatively, a few recent studies have used the spatial encoding provided by multichannel EPI data, and forward models trained on reference scans or prior information about the tissue structure to estimate dynamic off‐resonance in human fMRI.^25^, ^26^, ^27^ Specifically, Wallace et al.^27^ used multichannel free induction decay navigators followed by calibration of the complex navigator changes using a separate reference scan to estimate and correct the dynamic geometric distortion. Although this study successfully demonstrated geometric distortion correction, it is limited by requiring a separately acquired contrast‐matched reference scan and sequence modification.

To mitigate these issues, we propose to use the EPI reference navigator in order to estimate dynamic $B_{0}$ off‐resonance changes, without the need for any extra scans, sequence modification, or prior knowledge. This short navigator that samples the central line of k‐space three times at each shot following the imaging RF excitation pulse is already present in most typical EPI sequences and is widely used to correct the Nyquist ghosts caused by discrepancies between odd and even EPI lines.^28^, ^29^ Here we cast the linear off‐resonance perturbations as shifts in the navigator k‐space data, and used the generalized autocalibrating partial parallel acquisition (GRAPPA) operator formalism^30^ together with the spatiotemporal encoding provided by the multichannel EPI reference navigator to estimate spatially varying off‐resonance at every time frame. Dynamic off‐resonance estimates were then used to correct the k‐space data at each time frame in order to make it more consistent with the calibration data, effectively reducing the dynamic ghosting artifacts and also reducing geometric distortions as a byproduct. Performance of the proposed method is demonstrated by successfully estimating the manually introduced off‐resonance in phantom experiments, and improving reconstruction quality in simulated and in vivo SMS accelerated NHP fMRI.

## 2 METHODS

Our proposed method is based on estimating linear k‐space shifts in the EPI reference navigator data using GRAPPA operators. In the following sections, we first briefly review the concept of GRAPPA operators and their utility for generating arbitrary k‐space shifts, and then explain how they can be used in dynamic off‐resonance estimation and correction.

### 2.1 GRAPPA operators for k‐space shift

In GRAPPA, missing k‐space values are synthesized as a linear combination of acquired neighboring k‐space values from all channels, where the neighborhood used in the linear combination is determined by the geometry of the chosen interpolation kernel.^31^ Let $S\left(k\right)\in C^{J\times N}$ be the acquired data at some k‐space location $k=(k_{x},k_{y},k_{z})$, *J* be the number of receive channels, and *N* be the number of reconstructed k‐space points. In its simplest form, the GRAPPA reconstruction problem synthesizes each missing k‐space point using one acquired k‐space point from one adjacent phase‐encoding line, and can be cast as^32^

(1) $$\begin{array}{c} S\left(k_{y}+\Delta k_{y}\right)=G_{y}S\left(k\right) \end{array}$$

where $G_{y}\in C^{J\times J}$ is the matrix of GRAPPA weights obtained by calibration on fully sampled data. In this formalism, $G_{y}$ that maps each k‐space point to its adjacent point can be interpreted as an operator that shifts the k‐space data one location along the phase‐encoding direction. Recursive application of Equation (1) can be used to produce shifts by multiples of $\Delta k_{y}$

(2) $$\begin{array}{c} S\left(k_{y}+n\Delta k_{y}\right)={G_{y}}^{n}S\left(k\right)\;n\in Z \end{array}$$

arbitrarily small shifts can be achieved using fractional powers of the operator^30^

(3) $$\begin{array}{c} S\left(k_{y}+\delta k_{y}\right)={G_{y}}^{\delta k_{y}/\Delta k_{y}}S\left(k\right)\;\delta k_{y}\in R \end{array}$$

and shifts across multiple directions can be achieved by successive application of operators that were trained on different directions

(4) $$\begin{array}{c} S\left(k+\delta k\right)={G_{x}}^{\delta k_{x}/\Delta k_{x}}{G_{y}}^{\delta k_{y}/\Delta k_{y}}{G_{z}}^{\delta k_{z}/\Delta k_{z}}S\left(k\right) \end{array}$$

where $G_{x}$, $G_{y}$, and $G_{z}$ are the operators trained to perform shifts of one k‐space location along the read‐out, phase‐encoding, and slice‐encoding directions, respectively. Here, we trained GRAPPA operators $G_{x}$, $G_{y}$, and $G_{z}$ using the fully sampled calibration data and used them throughout the reconstruction. Note that the operator in the slice‐encoding direction is realisable only with 3D k‐space information. To address this issue in SMS EPI, we constructed a proxy 3D calibration dataset. To construct this dataset, individual slices from the original fully sampled calibration data were reconstructed and stacked along the slice axis. All but the encoded slices were set to zero, and the resulting 3D volume was transformed back to k‐space using a 3D Fourier transform.

### 2.2 Dynamic off‐resonance estimation and correction using EPI reference navigator

We used the fractional shift property of GRAPPA operators (Equation 4) and the three EPI reference navigator lines to estimate dynamic $B_{0}$ off‐resonance changes at each time‐frame of the fMRI time‐series. In awake NHP fMRI, the head is mechanically fixed but motion in body parts that are distant from the brain causes low spatial frequency off‐resonance changes.^10^ Therefore, assuming $\Delta \omega^{p}$ is the off‐resonance frequency during the acquisition of navigator lines at frame *p*, a first‐order approximation can be used to model that as dynamic perturbations around the off‐resonance at the reference frame (p=0)

(5) $$\begin{array}{c} \Delta \omega^{p}=\Delta \omega^{0}+2\pi br \end{array}$$

where $b=(b_{x},b_{y},b_{z})$ is the vector of coefficients, and $r={\left(x,y,z\right)}^{T}$ is the spatial location.

Letting $s_{j}^{p}\left(t\right)$ to be the acquired navigator signal from channel *j* at time‐frame *p*, the signal in the presence of off‐resonance can be expressed as

(6) $$\begin{array}{c} s_{j}^{p}\left(t\right)=\int C_{j}\left(r\right)\rho \left(r\right)e^{‐i2\pi \mathrm{kr}}e^{‐i\Delta \omega^{p}\left(r\right)t}dr \end{array}$$

where $C_{j}$ is the sensitivity profile for channel *j* and ρ is the object magnetization. Using Equation (5), the navigator signal can be expressed as

(7) $$\begin{array}{c} s_{j}^{p}\left(t\right)=\int C_{j}\left(r\right)\rho \left(r\right)e^{‐i2\pi [k+b(t)]r}e^{‐i\Delta \omega^{0}t}dr \end{array}$$

**FIGURE 2:**
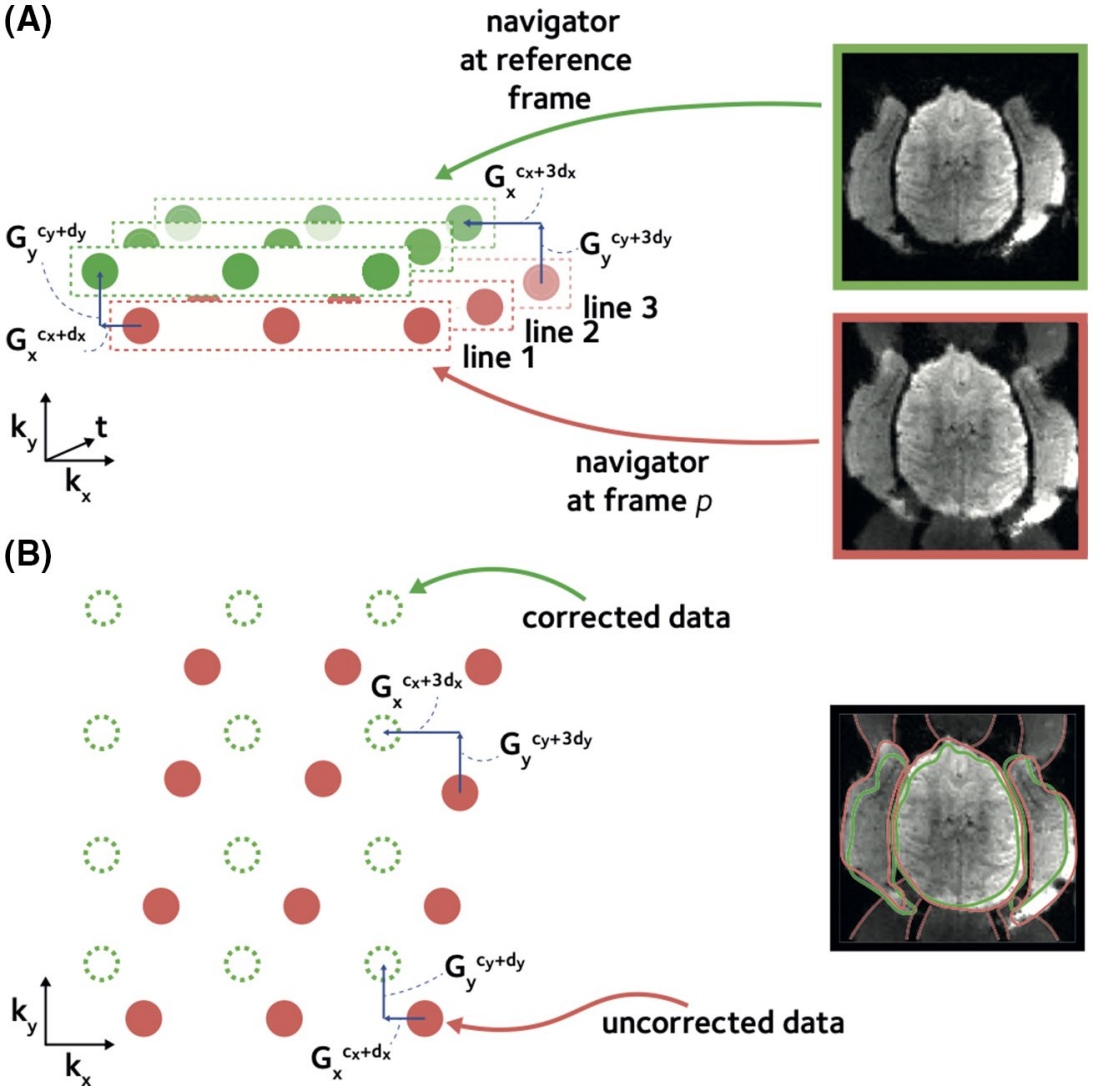
Dynamic off‐resonance estimation and correction. Spatially linear off‐resonance perturbations can be cast as linear shifts in k‐space data. (A) To estimate the off‐resonance perturbation, the EPI reference navigator data at each time frame were compared to the navigator data from a reference frame. Linear shifts between corresponding navigator lines were estimated using GRAPPA operators, accounting for the different echo times of the consecutive navigator lines. (B) The estimated linear shift coefficients that reflect the off‐resonance perturbation were used to correct the EPI data at each time frame. This procedure reduces the ghosting artifacts and geometric distortion (red outline), yielding an improved reconstruction (green outline)

Thus, the navigator data acquired at frame *p* can be modeled as a shifted version of the navigator data at frame 0

(8) $$\begin{array}{c} S_{l}^{p}\left(k\right)=S_{l}^{0}{(k+b}_{l})\;l\in \left\{1,2,3\right\} \end{array}$$

where $S_{l}^{p}\left(k\right)\in C^{M\times J}$ is the *l*th navigator line at frame *p*, and *M* is the total number of k‐space columns. Note that because the phase induced by the off‐resonance in three consecutive navigator lines varies due to different echo times, the shifts for the three lines are distinct in Equation (8) (see Figure 2A). Assuming a constant echo spacing between consecutive EPI line acquisitions the shifts in consecutive navigator lines increase linearly

(9) $$\begin{array}{c} b_{l}=c+ld\;l\in \left\{1,2,3\right\} \end{array}$$

The first‐order off‐resonance perturbation at frame *p* can be modeled using the linear shifts in the three navigator lines compared to the navigator data at the reference frame

(10) $$\begin{array}{c} \left[\begin{array}{c} S_{1}^{p}\\ S_{2}^{p}\\ S_{3}^{p} \end{array}\right]=\left[\begin{array}{ccc} {G_{x}}^{c_{x}+d_{x}}{G_{y}}^{c_{y}+d_{y}}{G_{z}}^{c_{z}+d_{z}} & 0 & 0\\ 0 & {G_{x}}^{c_{x}+2d_{x}}{G_{y}}^{c_{y}+2d_{y}}{G_{z}}^{c_{z}+2d_{z}} & 0\\ 0 & 0 & {G_{x}}^{c_{x}+3d_{x}}{G_{y}}^{c_{y}+3d_{y}}{G_{z}}^{c_{z}+3d_{z}} \end{array}\right]\left[\begin{array}{c} S_{1}^{0}\\ S_{2}^{0}\\ S_{3}^{0} \end{array}\right] \end{array}$$

resulting in a model with six unknown off‐resonance coefficients and 3M×J constraints, where $c=[c_{x},c_{y},c_{z}]$ contribute to the offset off‐resonance, and $d=[d_{x},d_{y},d_{z}]$ contribute to the time‐varying component of off‐resonance. The model in Equation (9) captures the spatially first‐order time‐independent field changes and cannot explain the contributions by the zeroth‐order changes (i.e., global frequency variations). However, it should be noted that correcting for global frequency variation is automatically performed in most typical EPI sequences, and thus is ignored in this formulation.

The coefficients c and d at each frame were estimated by solving the problem in Equation (10) using least squares. Note that the navigators are fully sampled along the readout direction and provide more information in this direction compared to the phase‐encoding direction. Specifically, we noted that in frames with unusually high off‐resonance the estimated $d_{y}$ coefficient could be less accurate. To address such inaccuracies, an additional refinement step was performed at each frame. In the refinement step, the estimated coefficients were first used to yield an intermediate image. Then a grid search was performed by perturbing the $d_{y}$ coefficient around the initial estimate to maximize the product‐moment correlation coefficient between the intermediate image and the fully sampled reference image. Finally, the estimated off‐resonance coefficients were used to correct the EPI data at each time frame.

A python implementation of the proposed method is available at https://github.com/shahdloo/nhp_recon.

### 2.3 Experiments

#### 2.3.1 Manual shim manipulation

To validate the performance of the proposed method in estimating dynamic off‐resonance, a bottle phantom was scanned on a horizontal 3T scanner using a 15‐channel custom NHP receive coil (RAPID Biomedical, Rimpar, Germany) and the CMRR multiband gradient‐echo (GRE) EPI sequence^3^, ^4^ with parameters: TE/TR = 30/2000 ms, FA = 90, FOV = 192, 1.5 mm isotropic resolution, 24 slices, multiband acceleration factor MB=2. To mimic dynamic off‐resonance changes, separate scans were performed where first‐order shim terms were manually adjusted up to ±20μ T/m in 5μ T/m increments across acquisitions. The proposed method was then used to estimate the changes in linear shim terms, using the navigator data only.

#### 2.3.2 Dynamic off‐resonance correction in vivo

To examine the performance of the proposed method in estimating and correcting the dynamic off‐resonance effects in vivo, 2D fMRI data from an awake behaving Macaque monkey using both in‐plane and SMS acceleration were collected. All procedures were conducted under the relevant animal care and use licenses from the UK Home Office in accordance with the UK Animals Act 1986 (Scientific Procedures) and with the European Union guidelines (EU Directive 2010/63/EU). The animal was head‐fixed in the sphinx position in an MRI‐compatible chair (Rogue Research). Data were acquired using the CMRR multiband GRE‐EPI sequence with parameters: TE/TR = 30/2210 ms, FA = 90, FOV = 128, 1.25 mm isotropic resolution, 40 slices, MB=2, in‐plane acceleration factor R=2, 50 frames, using the same 15‐channel NHP receive coil. Single‐band data and fully sampled calibration data were acquired along with the functional data as separate parts of the imaging sequence. To minimise the motion effects during acquisition of the calibration data, Fast Low‐angle Excitation Echo‐planar (FLEET) method was used, so segments associated with the same slice were acquired sequentially in time very quickly, with little noticeable artifact. Standard Nyquist ghost correction and dynamic zeroth‐order B0 correction were applied on all reconstructions prior to off‐resonance estimation and correction. Standard ramp‐sampling was used.^35^ Note that this could confound the off‐resonance estimation when the whole navigator line is used in off‐resonance estimation. However, it should be noted that central part of the navigator data which is least affected by the ramp‐sampling contains the most relative signal power and dominates the noisy outer regions. This has been verified by comparing the off‐resonance estimation and correction performance using the whole navigator lines versus using only the central parts. Results were not noticeably different between the two alternatives (not shown), and hence the full navigator lines were used throughout the study.

The single‐band data were used to simulate a generic accelerated fMRI data with in‐plane and SMS undersampling (MB=2, R=2) corrupted by dynamic off‐resonance. Random first‐order off‐resonance perturbations (0 ± 20 Hz, mean ± std across the field of view) were assumed at each frame separately for each slice pair, and were used to retrospectively warp the single‐band data using multifrequency interpolation.^36^ Data were then summed across each slice pair. The proposed method was used to estimate the simulated dynamic off‐resonance at each frame, and the estimates were used to correct the data.

Finally, to evaluate the proposed method in vivo, dynamic off‐resonance was estimated for the whole prospectively undersampled fMRI series, and the data were corrected with the proposed method prior to unaliasing reconstruction using the split‐slice GRAPPA algorithm.^34^

### 2.4 Quantitative measures

Shannon’s entropy was used to measure ghosting artifact level in the images, as suggested by previous studies.^37^, ^38^ Entropy was calculated as

(11) $$\begin{array}{c} E\left(I\right)=‐\sum_{k=1}^{N}I_{k}\log_{2}\left(I_{k}\right), \end{array}$$

where $I_{k}$ is pixel magnitude and *N* is the number of image pixels.

To quantify geometric distortion, normalized root mean squared error (nRMSE) compared to the reconstructed single‐band reference was used. nRMSE was calculated as

(12) $$\begin{array}{c} \text{nRMSE}\left(I,I_{r}\right)=\frac{\sqrt{\text{mean}\left({\left(I‐I_{r}\right)}^{2}\right)}}{\max(I)‐\min(I)}, \end{array}$$

where *I* is the reconstructed image and $I_{r}$ is the reference image. Note that since the reference image is reconstructed from the undersampled single‐band data, the nRMSE measured here would be slightly overestimated relative to an absolute ground truth.

**FIGURE 3:**
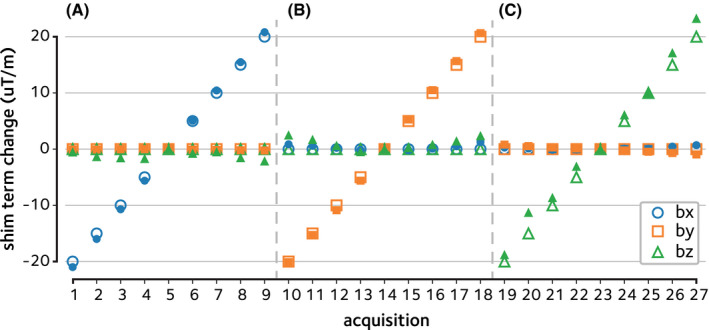
Estimation of linear shim changes in phantom. Dynamic off‐resonance changes were induced in a bottle phantom by manually modifying the linear shim terms across acquisitions. The EPI reference navigator data were then used to estimate the linear shim term changes across (A) *x*, (B) *y*, and (C) *z* directions in the range ±20μ T/m. The estimated shim term changes (filled markers) are in good agreement with the ground truth (empty markers)

To assess the effect of image quality improvements on the fMRI time series, temporal signal to noise ratio (tSNR) was compared between reconstructions, after masking out the non‐brain voxels. While tSNR does not fully characterize the impact of temporally stable ghosting or aliasing artifacts, the metric is useful for capturing dynamic image instabilities that tend to be the main source of ghosting artifacts in awake NHP fMRI. tSNR in pixel *k* was calculated as

(13) $$\begin{array}{c} \text{tSNR}\left(I_{k}\right)=\frac{\text{mean}(I_{k})}{\text{std}(I_{k})}. \end{array}$$

## 3 RESULTS

The performance of the proposed method in estimating manual shim changes in the phantom is shown in Figure 3. Linear shim term changes in the range of ±20μT/m are accurately estimated with absolute errors of 0.67±0.14μ T/m (mean ± sem across acquisitions).

**FIGURE 4:**
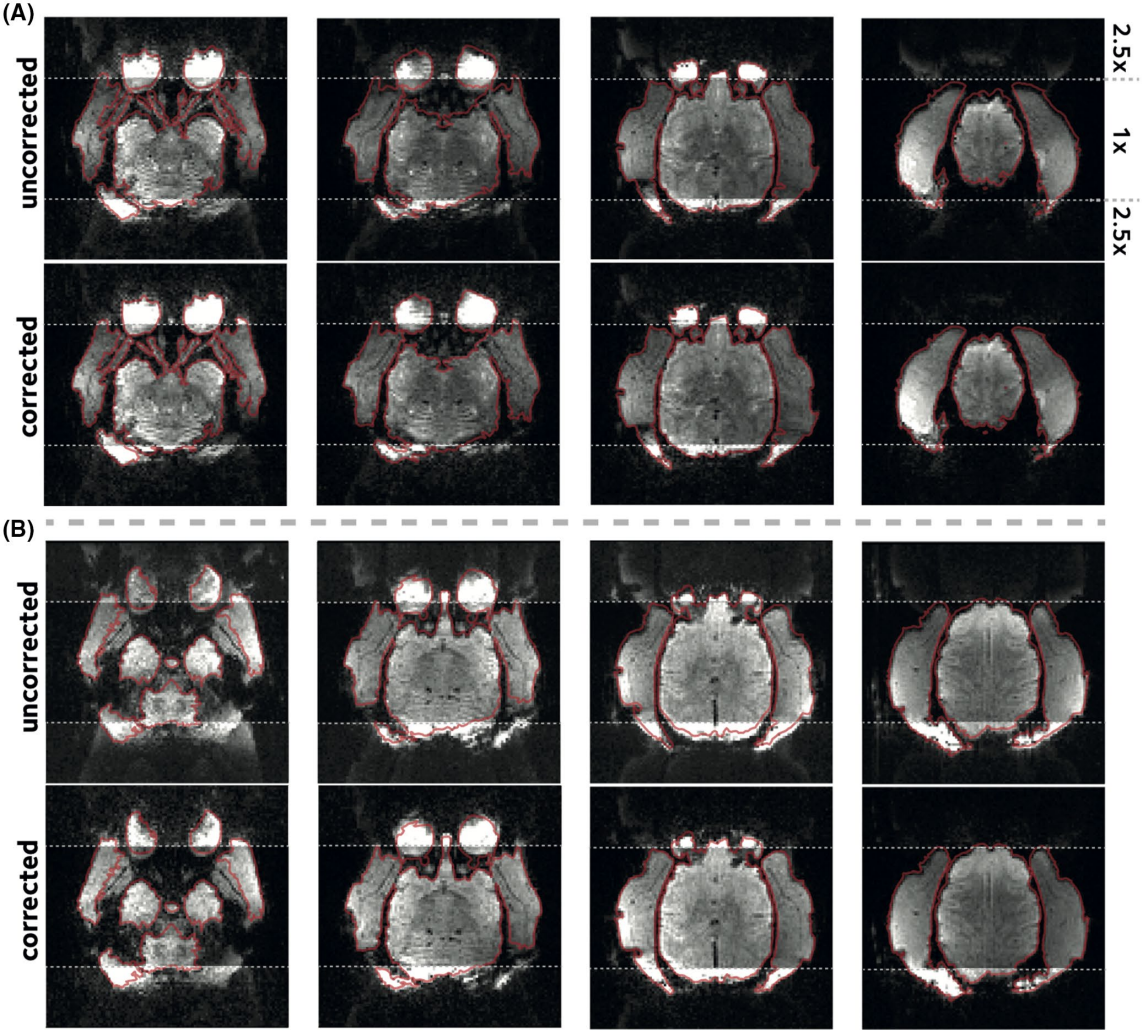
Reconstruction of the in vivo EPI acquisition. (A) SMS accelerated in vivo data were simulated using the single‐band k‐space data by assuming dynamic off‐resonance perturbations. Calibration inconsistency due to off‐resonance perturbation causes ghosting artifacts and geometric distortions. EPI reference navigator data were used to estimate and correct the simulated dynamic off‐resonance. Standard Nyquist ghost correction and dynamic zeroth‐order B0 correction were applied on both corrected and uncorrected image series prior to off‐resonance estimation and correction. Images from four different slices are shown in columns. The display window in the top and bottom quarter of images are saturated to better show the ghosting artifacts. The red outline shows the object boundary in the undistorted single‐band reference image. (B) Prospectively SMS accelerated in vivo acquisition was corrected and reconstructed using the proposed method. Formatting is identical to panel A. Estimating and accounting for the dynamic off‐resonance yields significantly reduced ghosting artifacts and geometric distortion

Next, the proposed method is demonstrated in reconstructions of the in vivo data. Figure 4A shows the reconstructions of the retrospectively accelerated data with simulated dynamic off‐resonance (difference images shown in Supporting Information Figure S1). Significant ghosting artifacts and geometric distortions that are originally present in the data are successfully attenuated using the proposed approach. Mean image entropy was reduced from 2.19±0.12 kbits (mean ± sem across time‐frames) in the online reconstruction to 1.72±0.10 kbits in the reconstruction with the proposed method, a 21% decrease, indicating a significant reduction in ghosting artifacts (bootstrap test, $p<10^{‐4}$). Moreover, nRMSE compared to the single‐band reference image was reduced from 9.43±0.09% in the online reconstruction to 6.31±0.12% using the proposed method, indicating a reduction in residual artifacts and geometric distortions.

**FIGURE 5:**
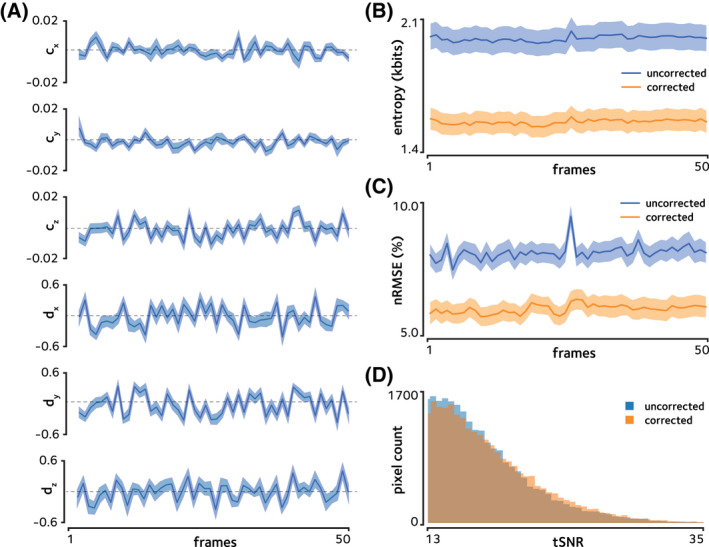
Estimated parameters and quantitative measurements for the in vivo EPI acquisition. (A) The six estimated off‐resonance model parameters are shown at each time time frame of the prospectively SMS accelerated in vivo acquisition. The display scaling of the offset terms $c_{x}$, $c_{y}$, and $c_{z}$ is magnified for easier visibility. Shaded areas show the standard error of the mean across slices. (B) Image entropy is shown at each time frame. Shaded areas show the standard error of the mean across slices. The proposed method decreases the mean entropy, indicating the decrease in ghosting artifacts. (C) Normalized root mean squared error compared to the single‐band reference image is shown. Shaded areas show the standard error of the mean across slices. Decreased geometric distortion achieved using the proposed method yields reduced nRMSE. (D) Histograms of tSNR is compared between the reconstructions. Only the tail of the histogram is shown to compare the distribution in pixels with highest tSNRs. The proposed method yields a histogram that is skewed to the right, indicating higher number of pixels with high tSNR

Figure 4B shows a comparison of the proposed reconstruction of the accelerated in vivo scan versus the online reconstruction provided as part of the CMRR multiband EPI sequence package,^3^ and quantitative comparisons are provided in Figure 5. Mean entropy was significantly reduced from 2.05±0.01 kbits in the online reconstruction to 1.63±0.01 kbits in the proposed method ($p<10^{‐4}$, Figure 5B). This 20% decrease is in line with the 21% decrease predicted by the simulations. Normalized RMSE was 8.49±0.04% in the online reconstruction, and was 6.16±0.02% in the proposed method (Figure 5C). These results show that ghosting or residual aliasing artifacts and geometric distortion in the prospectively SMS accelerated in vivo scan are considerably attenuated using the proposed method.

Finally, tSNR was found to be 13.51±0.03 (mean ± sem across pixels) in the online reconstruction, and 13.97±0.03 in the proposed method (Figure 5D), indicating an improvement in functional signal stability. It should be noted that since static or quasi‐static aliasing generates stable artifacts, removal of these artifacts would not significantly change tSNR, while clearly having a large impact on image quality and as such the tSNR enhancement reported here would not be on the same order as other reported image quality measures. That said, since BOLD signal variation in the image series is on the order of a few percent, even small changes in tSNR can have significant impact on BOLD statistics

To assess the effect of the refinement step on the performance of the method, an alternative reconstruction of the in vivo dataset without the refinement step was performed, yielding nRMSE of 6.86±0.03%, which is higher than that achieved by including the refinement step. However, this performance improvement comes at the cost of a slight increase in off‐resonance estimation time. Off‐resonance estimation time is 1.49±0.40s (mean ± std across slices and frames) by including the refinement step, and is 0.63±0.06s without the refinement step.

## 4 DISCUSSION

We have presented a new approach for estimating and correcting the linear dynamic $B_{0}$ off‐resonance perturbations in accelerated fMRI of awake behaving NHPs. Acceleration in NHP fMRI has significant impact on data quality and scan efficiency since in‐plane acceleration enables achieving higher spatial resolution and SMS acceleration increases statistical significance of the functional time‐series by increasing the acquired time samples collected at a set acquisition time.^39^ However, vigorous animal motion is unavoidable in imaging of awake and behaving NHPs, even with training. These dynamic effects degrade image quality by making the imaging data inconsistent with the calibration data used during the reconstruction. Our results in phantom, simulation, and in vivo experiments show that these effects can be accurately estimated and significantly attenuated using only the EPI reference navigator data that is included in most typical EPI sequences, or can be included with minor modifications if not already present. A key property of the proposed method is that it relies only on data acquired using conventional accelerated acquisitions, and does not require extensive sequence modification or lengthened scans to accommodate complex navigators or multiple echoes. As such, it is also possible to apply the proposed method retrospectively to previously acquired scans, if raw measurement data are available.

In awake NHP imaging, the reduced functional sensitivity is conventionally addressed by using contrast agents^40^, ^41^, ^42^, ^43^ or detecting and discarding data frames with excessive motion.^44^, ^45^ Using contrast agents biases the functional signal by changing the hemodynamic response functions, hindering direct comparison between findings in NHPs and human studies.^46^ Furthermore, detecting and discarding data frames with excessive motion requires extra monitoring hardware and leads to a reduction in the available temporal degrees of freedom. In contrast, the approach presented here can be used to enhance functional data quality in existing NHP acquisition protocols without sacrificing time‐points or requiring extra hardware.

While we have focused on demonstrating the proposed method in the context of NHP fMRI, this approach can be applied to other preclinical imaging applications that employ an accelerated EPI acquisition containing the EPI reference navigator, such as accelerated rodent fMRI. Moreover, this approach could have the potential to improve functional sensitivity in human neuroimaging applications in patients with uncontrolled movements such as in Parkinson’s disease, or in neonatal children.

Note that we have developed the problem with the assumption of having three navigator lines. This results in a model that can be cast as an overdetermined system of equations. However, the model is trivially generalizable to arbitrary number of navigator lines as long as they are acquired at each time frame. In general, at least two navigator lines would be needed in order to fully characterize the linear coefficients. Using a single line would limit our ability to distinguish B0‐related phase changes from time‐independent phase effects caused by sources such as the $B_{1}$ excitation field.

The GRAPPA kernels used in estimating the off‐resonance were trained on the constructed 3D proxy calibration dataset. This dataset would include more information at higher multiband factors. Thus, we expect that the estimated kernels to be more accurate at higher multiband factors, possibly leading to more accurate off‐resonance correction. Moreover, since the navigator data are acquired independently from the imaging data, there is no interaction between the in‐plane acceleration factor used in the actual images and the navigator‐derived field estimates.

Here, we have assumed first‐order spatial off‐resonance perturbations that have been shown to be a good approximation in imaging headposted NHPs.^10^ The theoretical k‐space navigator shifting framework in which we cast the problem relies on the first order perturbation assumption to treat the perturbations as additional linear encoding fields. The benefit of this is that we can use existing GRAPPA operator tools to facilitate off‐resonance estimation. One limitation of this work, however, is that the accuracy of the first‐order approximation can suffer with extreme body or head motion. A potential solution for such extreme cases is to use the proposed approach to provide a near optimal initial guess to be used in a nonlinear image‐based dynamic off‐resonance correction method, which would be an interesting direction for future research. It is also important to note that the quality of the proposed method depends on the accuracy of the estimated GRAPPA operators. An array with a small number or channels or with poorly arranged elements can result in inaccurate operator estimation due to insufficient coil sensitivity variation along one or more directions. Although the coil used here allowed us to estimate shifts up to Δk, using a less capable coil could have led to more residual artifacts due to suboptimal off‐resonance estimation and correction.

In conclusion, the method presented here enables more robust and reliable NHP imaging in accelerated acquisitions, with limited or no sequence or protocol changes, reducing the gap between what is possible with NHP protocols and state‐of‐the‐art human imaging.

## Supporting information


**FIGURE S1**: Error in the reconstruction of simulated in vivo data. Normalised error between the reconstructed and single‐band reference images, corresponding to reconstructions in Figure 4A, are shown. The proposed off‐resonance correction yields visibly reduced reconstruction errors.
