# Supplementary material for: Model‐based dynamic off‐resonance correction for improved accelerated fMRI in awake behaving nonhuman primates
Source: Magn Reson Med. 2022 Jan 26;87(6):2922–32. doi: 10.1002/mrm.29167 (PMC9306555; doi:10.1002/mrm.29167)
Supplement: Supplementary file 1 — FIGURE S1: Error in the reconstruction of simulated in vivo data. Normalised error between the reconstructed and single‐band reference images, corresponding to reconstructions in Figure 4A, are shown. The proposed off‐resonance correction yields visibly reduced reconstruction errors. [file MRM-87-2922-s001.pdf]

Supplementary Figures

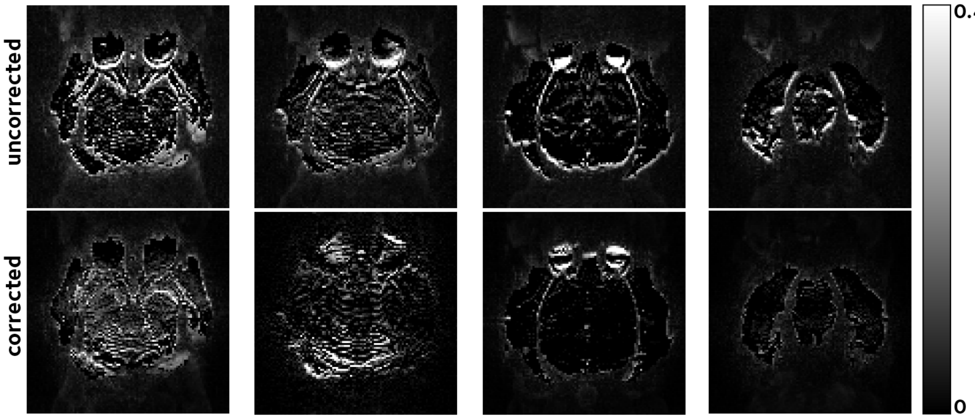

Supplementary Figure 1: **Error in the reconstruction of simulated *in vivo* data.**
Normalised error between the reconstructed and single-band reference images, corresponding to reconstructions in Fig. 4a, are shown. The proposed off-resonance correction yields visibly reduced reconstruction errors.
